# InterobServer AgreeMent in Pd‐l1 evaLuatIoN on cytoloGical samples—SAMPLING project: A multi‐institutional, international study

**DOI:** 10.1002/cncy.70003

**Published:** 2025-02-24

**Authors:** Gennaro Acanfora, Antonino Iaccarino, Bruna Cerbelli, Claudio Di Cristofano, Claudio Bellevicine, Massimo Barberis, Emanuela Bonoldi, Lukas Bubendorf, Andreas Calaminus, Severo Campione, Sule Canberk, Alberto Cavazza, Giorgio Cazzaniga, Obinna Chijioke, Eduardo Clery, Albino Eccher, Marianne Engels, Vincenzo Fiorentino, Paolo Graziano, Izidor Kern, Ivana Kholova, Jari Laatta, Tania Labiano, Martina Leopizzi, Maria D. Lozano, Rita Luis, Elisabetta Maffei, Alessandro Marando, Maurizio Martini, Elisabetta Merenda, Marco Montella, Allan Argueta Morales, Michiya Nishino, Fabio Pagni, Paul Hofman, Angelina Pernazza, Sinchita Roy‐Chowdhuri, Mauro Saieg, Spasenija Savic Prince, Momin T. Siddiqui, Tajana Stoos‐Veic, Margareta Strojan Fležar, Dinka Sundov, Paul VanderLaan, Danijela Vrdoljak‐Mozetič, Pio Zeppa, Giancarlo Troncone, Elena Vigliar

**Affiliations:** ^1^ Department of Public Health University of Naples “Federico II” Naples Italy; ^2^ Department of Medico‐Surgical Sciences and Biotechnology Polo Pontino Sapienza University Roma Italy; ^3^ Advanced Molecular Diagnostics Istituto Europeo di Oncologia IRCCS Milano Italy; ^4^ Niguarda Cancer Center Niguarda Hospital Milano Milano Italy; ^5^ Institute of Medical Genetics and Pathology University Hospital Basel Basel Switzerland; ^6^ Institut fur Pathologie Uniklinik Koln Cologne Germany; ^7^ Department of Advanced Diagnostic‐Therapeutic Technologies and Health Services Section of Anatomic Pathology A. Cardarelli Hospital Naples Italy; ^8^ Institute of Molecular Pathology and Immunology of the University of Porto (Ipatimup) Cancer Signalling and Metabolism Research Group of Instituto de Investigação e Inovação em Saúde (i3S) Porto Portugal; ^9^ Pathology Unit Azienda USL/IRCCS di Reggio Emilia Reggio Emilia Italy; ^10^ Department of Medicine and Surgery Pathology IRCCS Fondazione San Gerardo dei Tintori University of Milano‐Bicocca Milan Italy; ^11^ Pathology Unit AOU University of Campania “Luigi Vanvitelli” Naples Italy; ^12^ Department of Medical and Sciences for Children and Adults University of Modena and Reggio Emilia University Hospital of Modena Modena Italy; ^13^ Department of Human Pathology in Adult and Developmental Age “Gaetano Barresi” University of Messina Messina Italy; ^14^ Department of Radiology, Oncology and Pathology University of Rome Sapienza Rome Italy; ^15^ Cytology and Pathology Laboratory University Clinic of Respiratory and Allergic Diseases Golnik Golnik Slovenia; ^16^ Faculty of Medicine and Health Technology Tampere University Tampere Finland; ^17^ Pathology Fimlab Laboratories Tampere Finland; ^18^ Pathology Department University Hospital of Navarra Pamplona Spain; ^19^ Pathology Department University Clinic of Navarra Pamplona Spain; ^20^ Department of Pathology Unidade Local de Saúde São José Lisbon Portugal; ^21^ Pathology Unit University Hospital of Salerno Salerno Italy; ^22^ Department of Pathology Beth Israel Deaconess Medical Center and Harvard Medical School Boston Massachusetts USA; ^23^ Université Côte d'Azur Nice France; ^24^ FHU OncoAge IHU RespirERA Laboratory of Clinical and Experimental Pathology Pasteur Hospital Nice France; ^25^ Department of Pathology The University of Texas MD Anderson Cancer Center Houston Texas USA; ^26^ Santa Casa Medical School Sao Paulo Brazil; ^27^ Department of Pathology and Laboratory Medicine Weill Cornell Medicine/New York Presbyterian Hospital New York New York USA; ^28^ Pathology and Cytology Department University Hospital Dubrava University of Zagreb School of Medicine Zagreb Croatia; ^29^ Institute of Pathology Medical Faculty University of Ljubljana Ljubljana Slovenia; ^30^ Department of Pathology Forensic Medicine and Cytology University Hospital of Split University of Split School of Medicine Split Croatia; ^31^ Department of Pathology and Cytology Clinical Hospital Centre Rijeka Rijeka Croatia

**Keywords:** cell block, cytology, interobserver variability, PD‐L1, tissue microarray

## Abstract

**Introduction:**

The aim of this project is to assess interobserver agreement for programmed death‐ligand 1 (PD‐L1) scoring on of non–small cell lung cancer (NSCLC) on cytological specimens in a large‐scale multicenter study, by exploiting the cell block‐derived tissue microarray (cbTMA) approach.

**Methods:**

A total of 65 cell blocks (CB) diagnosed as NSCLC were retrospectively collected and selected for TMA preparation. Hematoxylin–eosin and PD‐L1 stained slides were digitized and uploaded on a free web sharing platform. Participants were asked to provide PD‐L1 assessment by using the clinically relevant cutoff of tumor proportion score (TPS) (<1%; 1%–49%; >50%). Interobserver agreement was calculated using Fleiss’s κ.

**Results:**

Of 65 CBs, 11 were deemed not suitable; therefore, an overall number of 54 cores were used for the preparation of four TMAs. A total of 1674 evaluations were provided by 31 cytopathologists from 21 different institutions in nine countries. The statistical analysis showed a moderate overall agreement (κ = 0.49). The highest agreement was achieved in the TPS >50% category (κ = 0.57); moderate agreement was observed in TPS <1% category (κ = 0.51) and the lowest κ value was obtained for TPS 1%–49% category (k = 0.32).

**Conclusions:**

The overall moderate agreement observed showed that there is still room for improvement in inter‐pathologist agreement for PD‐L1 evaluation on cytological samples, highlighting the need for standardization in sample preparation, focused training in PD‐L1 evaluation on cytological material, and the integration of machine learning tools to improve interobserver consistency.

## Abstract 2

The overall moderate agreement observed highlights that improvement is still needed in inter‐pathologist agreement for programmed death‐ligand 1 (PD‐L1) evaluation on cytological samples. Sample preparation standardization, focused training in PD‐L1 evaluation on cytological material, its inclusion in External Quality Assessment, and machine learning‐derived image analysis tools could improve interobserver agreement.

## INTRODUCTION

Currently, standard treatments for several cancer types include monoclonal antibodies targeting the programmed death 1 (PD‐1)/programmed death ligand‐1 (PD‐L1) axis, due to their effectiveness. Nevertheless, given that only a small subset of patients benefit from immune checkpoint inhibitors (ICIs), which may also trigger immune‐related adverse events, the interest in identifying predictive biomarkers is not surprising.^1^ Indeed, the role of tumor mutational burden (TMB) in accessing to immunotherapy seems to be promising.^2^, ^3^

Despite investigations into several biomarkers expressed by tumor cells and their microenvironment, the immunohistochemical (IHC) evaluation of PD‐L1 still remains one of the most important predictive biomarkers for PD1/PD‐L1 immunotherapy.^1^, ^4^

To ensure the accessibility of immunotherapy to advanced cancer patients, for which the diagnostic material is often represented by small tissue samples, many efforts have been performed to implement and validate PD‐L1 IHC scoring on cytological material, such as in non–small cell lung cancer (NSCLC). This is primarily performed by assessing the tumor proportion score (TPS), which is the percentage of tumor cells that show any intensity of membranous PD‐L1 expression. Moreover, PD‐L1 testing on small samples is being increasingly performed due to the recent indication of immunotherapy in the neoadjuvant setting.^5^

Unfortunately, PD‐L1 expression is widely considered a less‐than‐perfect biomarker, affected by biological, preanalytical, and analytical issues.^6^, ^7^, ^8^ Notably, real‐world data show a large amount of variation in PD‐L1 positivity in NSCLC patients among laboratories.^8^, ^9^ The largest variation seemed to occur mostly at the 1% cutoff threshold for PD‐L1 staining, on both histological and cytological samples; although the use of cytological material for PD‐L1 testing has also shown challenges even at the 50% cutoff threshold.^9^ These findings suggest that the use of cytological material may significantly contribute to the general interlaboratory variation in assessing PD‐L1 positivity, due not only to pre‐analytical variables in specimens processing but also to the interpretation of PD‐L1 staining on cytological preparations, which may be more challenging than traditional histologic sections. However, several studies showed similar cytohistological concordance rate for 1%–49% and >50% PD‐L1 staining cutoff in matched NSCLC samples.^10^

Data on interobserver agreement for scoring of PD‐L1 TPS on cytological material have been limited to few studies involving limited number of pathologists and/or samples.^11^, ^12^, ^13^, ^14^, ^15^, ^16^, ^17^

Although tissue microarrays (TMA) have been used in conducting interobserver concordance studies, as well as multiplex analysis and validation of tumor markers on histological material, this approach is rarely applied to cytological specimens. In fact, few studies showed that TMAs can effectively be used with cell block (CB) specimens, especially those with higher cellularity, accurately reflecting the properties of the original CB.^18^, ^19^, ^20^, ^21^ Recently, we evaluated the technical feasibility of PD‐L1 assessment on CB‐derived TMA (cbTMA), providing a proof of concept that this approach could be used for biomarker testing validation on cytological material.^22^ In this study, we exploited the cbTMA approach to assess interobserver agreement for PD‐L1 scoring on cytological specimens in a large‐scale multicenter study.

## MATERIALS AND METHODS

A total of 65 CBs diagnosed as NSCLC (*n* = 36 primary lung tumors and *n* = 29 pleural effusions) were retrospectively collected and selected for the preparation of TMAs by four participating institutions (University of Naples Federico II [Italy], Antonio Cardarelli Hospital, Naples [Italy], Sapienza University of Rome [Italy], and Clínica Universidad de Navarra, Pamplona [Spain]).

In each center, routine samples are fixed in 10% neutral buffered formalin for 12–48 hours and embedded in paraffin. Cell block preparation protocols included simple sedimentation (*n* = 28), self‐clotting method (*n* = 24), and Shandon Cytoblock Cell Block Preparation System (Thermo Fisher Scientific Inc, Waltham, Massachusetts) according to the manufacturer's instructions (*n* = 14). One case of placental tissue was also added to the cases series as positive control for PD‐L1 immunostaining. TMA were prepared at Sapienza University of Rome; core biopsies of 1 mm in diameter were taken from each paraffin‐embedded cell block (donor block) and arrayed into a new recipient paraffin block (45 mm × 20 mm) using the ATA‐100 Chemicon International System. Reference cores of human placental tissue included in each TMA were used as positive control for PD‐L1 immunostaining. Sections (4‐μm thick) cut from TMA blocks were used for hematoxylin–eosin stain (H & E) and PD‐L1 IHC and carried out at the University of Naples Federico II, with the companion diagnostic kit SP263 assay (Ventana), following the manufacturer's instructions.

All stained slides were digitized with Hamamatsu Nanozoomer mod.RS 2.0 at ×40 magnification and uploaded on the free web sharing platform PathPresenter (https://www.pathpresenter.com/); a link to a digital slide collection was generated and shared with participants to visualize and score the cases.

Participants provided information on the institution type they belonged, the years of experience in cytopathology, the type of training received for PD‐L1 evaluation, the median volume per month of PD‐L1 IHC on lung cancer cytological samples, PD‐L1 antibody clone used in their institution, and TPS tier system used in their country (e.g., three‐tier approach of <1%, 1%–49%, and >50% or the two‐tier approach of <1% and >1%). In fact, although, the threshold of eligibility to immunotherapy is TPS >1%, the further stratification could impact on the choice of combination therapies.

Participants were asked to provide PD‐L1 assessment by using the TPS, selecting a value (<1%, 1%–49%, or ≥50%) from a drop‐down menu in an Excel spreadsheet. Participants were also asked to assess specimen adequacy according to the criteria for routine clinical practice (<100 cells vs. >100 cells) for each case. Although TPS cannot be assessed without a minimum of 100 cancers cells, taking into account the expected paucicellularity related to the use of CB‐derived cores, TPS score was required even for samples showing only a few neoplastic cells. For samples with total absence of neoplastic cells, participants could designate the case as “No neoplastic cells.” Specimens with preparation issues (e.g., folds in the TMA tissue) precluding TPS assessment could be designated by the participants as "Not evaluable.”

To test the overall interobserver agreement , Fleiss’s κ for multiple raters was calculated for the two‐tier and three‐tier TPS approach. The overall agreement was also assessed for the adequacy evaluation according to the routine criteria (<100 cells vs. >100 cells). Moreover, the overall interobserver agreement was also evaluated only for adequate samples according to the routine criteria (>100 cells). Statistical analyses were conducted using R Studio 2023.03.01 + 446 software (R Core Team, Vienna, Austria). The strength of association (agreement) was categorized as follows: 1.00, perfect agreement; 0.80–0.99, almost perfect agreement; 0.60–0.79, substantial agreement; 0.40–0.59, moderate agreement; 0.20–0.39, fair agreement; 0.0–0.19, poor agreement; and less than 0, no agreement.

All patient data were collected anonymously and written informed consent, as part of the routine diagnosis and treatment procedures, was obtained from patients or their guardians according to the Declaration of Helsinki and the study adhered to Good Clinical Practice guidelines.

## RESULTS

**FIGURE 1:**
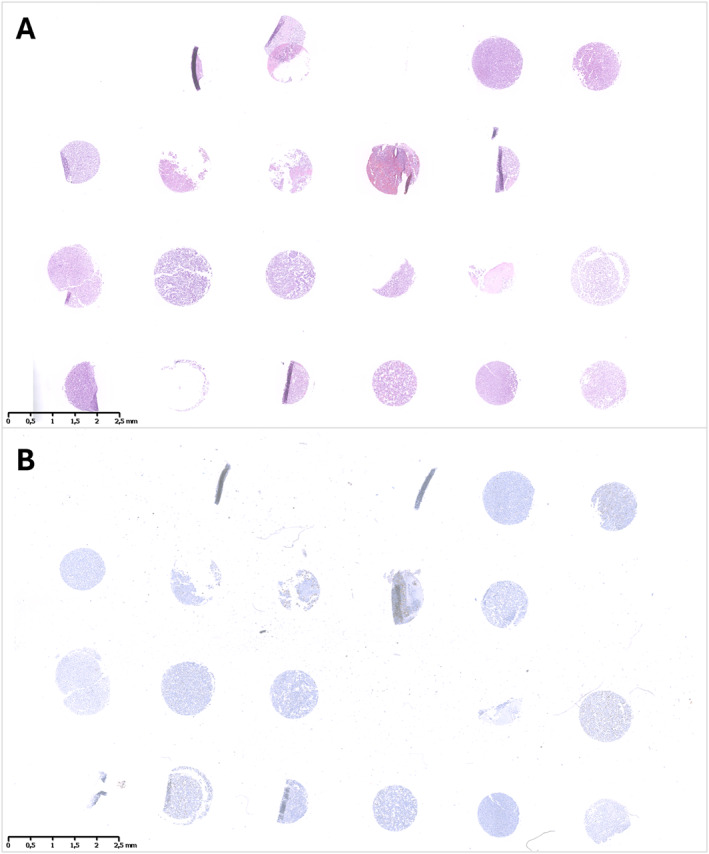
Example of TMA whole slide section: H & E stained slide (A) and immunocytochemistry for PD‐L1 (SP263) (B). PD‐L1 indicates programmed death‐ligand 1; TMA, tissue microarray.

Of 65 CBs, 11 were deemed not suitable; therefore, an overall number of 54 cores were used for the preparation of four TMAs. However, due to the different cellularity of selected samples, the number of cores was variable between TMAs, containing respectively *n* = 23, *n* = 23, *n* = 2, and *n* = 6 cores (Figure 1).

**FIGURE 2:**
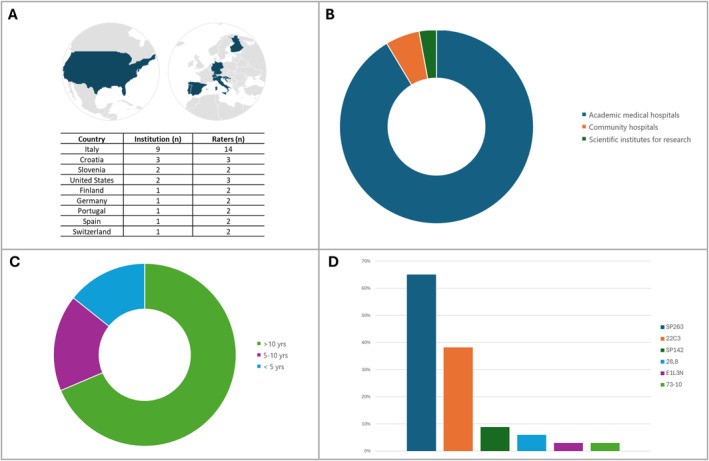
General data from participant raters. (A) Geographical distribution. (B) Institution type. (C) Years of experience in cytology. (D) PD‐L1 antibody clones used in home institution. PD‐L1 indicates programmed death‐ligand 1.

Thirty‐one cytopathologists from 21 different institutions in nine different countries (Italy, Croatia, Slovenia, United States, Finland, Germany, Portugal, Spain, and Switzerland) independently analyzed and scored the 54 cores, for a total of 1674 evaluations (Figure 2A).

A total of 91.4% of participants came from academic medical hospitals, 5.7% from community hospitals, and 2.9% from scientific institutes for research (Figure 2B). Cytopathology experience of the participants was reported as follows: greater than 10 years (68.6%), 5–10 years (17.1%), and less than 5 years (14.3%) (Figure 2C). Formal training for PD‐L1 assessment was received by *n* = 16 raters, training by trained by *n* = 14 raters, and online training by *n* = 7 raters. More than one training type was reported by *n* = 5 raters.

The median volume per month of PD‐L1 immunocytochemistry on lung cancer cytological samples was *n* = 16 (ranged between *n* = 2 and *n* = 75). As far as the types of PD‐L1 antibody clones, the results indicated the following usage: 65% SP263, 38.2% 22C3, 8.8% SP142, 5.9% 28‐8, 2.9% E1L3N, and 2.9% 73‐10 (Figure 2D).

The most TPS tier approach was the three‐tier system (93%, followed by the two‐tier system (3%). One rater (3%) reported a six‐tier approach (<1%, 1%–5%, 5%–10%, 10%–25%, 25%–50%, and >50%).

Interobserver variability analysis with the Fleiss κ methodology showed moderate overall agreement for the two‐tier TPS approach among the 31 raters (κ = 0.57); in particular κ = 0.51 was achieved in the <1% category and κ = 0.58 was achieved in the >1% category. Interobserver analysis across all TPS categories showed overall moderate agreement (κ = 0.49). The highest agreement was achieved in the TPS≥50% category (κ = 0.57); similarly, a moderate agreement was obtained in the TPS<1% category (κ = 0.51). Conversely, the lowest κ value was obtained within TPS 1%‐49% category (κ value 0.32, fair agreement) (Table 1). The overall interobserver agreement for adequate samples according to the routine criteria (>100 cells) was κ = 0.49.

**TABLE 1:** Interobserver variability across all TPS categories.

Category	κ value	Category	κ value	*p*	Interpretation
TPS <1%	0.51	TPS <1%	0.51	<.01	Moderate agreement
TPS >1%	0.58	TPS 1%–49%	0.32	<.01	Fair agreement
		TPS ≥50%	0.57	<.01	Moderate agreement
All categories	0.57	All categories	0.49	<.01	Moderate agreement

Abbreviation: TPS, tumor proportion score.

**FIGURE 3:**
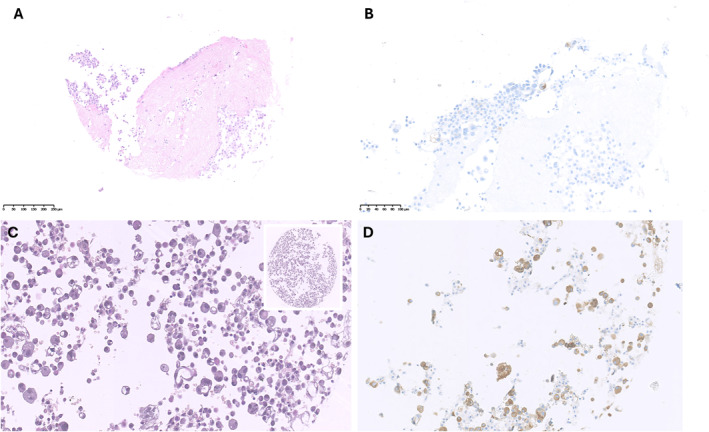
Core B16. (A) H & E staining. (B) PD‐L1 IHC showing few neoplastic cell exhibiting clear membrane positivity in the context of a neoplastic population with focal and faint nonspecific staining. Core A1: (C) H & E staining; (D) PD‐L1 IHC showing numerous scattered cells with nonspecific cytoplasmatic staining. IHC indicates immunohistochemical; PD‐L1, programmed death‐ligand 1.

**FIGURE 4:**
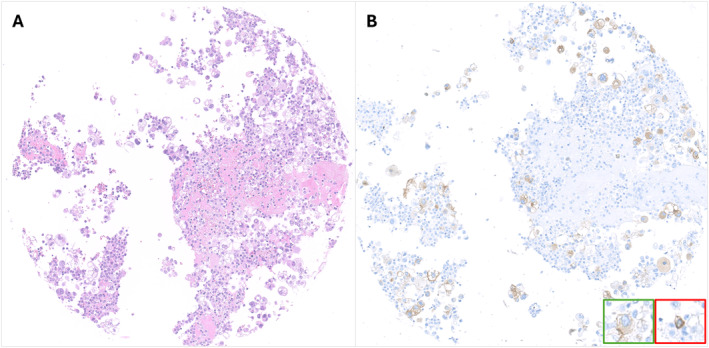
Core B8. (A) H & E staining; (B) PD‐L1 IHC showing cells with focal clear specific membranous staining, nonspecific cytoplasmatic staining and diffuse cytoplasmatic staining in macrophages. Green close‐up: nonspecific cytoplasmatic staining. Red close‐up: focal clear specific membranous staining. IHC indicates immunohistochemical; PD‐L1, programmed death‐ligand 1.

**FIGURE 5:**
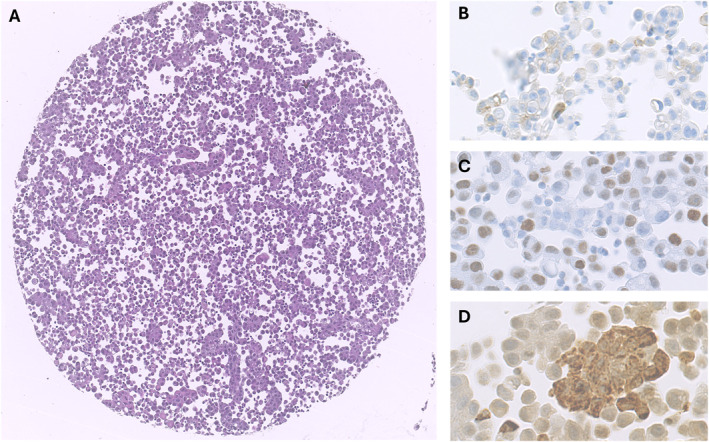
Core A7. (A) H & E staining showing a well differentiated adenocarcinoma: neoplastic cells are both in groups and scattered cells with interspersed histocytes. (B) PD‐L1 IHC showing specific membrane signal in discohesive cells. (C) TTF1 immunocytochemistry. (D) CD68 immunocytochemistry. IHC indicates immunohistochemical; PD‐L1, programmed death‐ligand 1.

Review of cases with high variability in TPS assessment revealed three types of interpretive pitfalls: 1) nonspecific cytoplasmatic staining in neoplastic and/or nonneoplastic cells; 2) membranous staining in nonneoplastic cells; and 3) positivity interpretation in discohesive samples (Table 2). As an example, the raters’ assessment of core B16 was distributed between TPS <1% and 1%–49%. The scarcity of neoplastic cells exhibiting clear membrane positivity in the context of a neoplastic population showing focal and faint nonspecific staining likely led to the discordant TPS call (Figure 3A,B). Core A1 is an example of a case for which the markedly divergent TPS scores (<1% vs. >50%) among raters could be attributed to the presence of numerous scattered cells showing nonspecific cytoplasmatic staining (Figure 3C,D). Two cases had TPS assessments distributed across all three categories (core B8 and core A7). Core B8 showed an adenocarcinoma with cells arranged in groups and dispersed with focal clear specific membranous staining and nonspecific cytoplasmatic staining. Diffuse cytoplasmatic staining in macrophages was also observed (Figure 4). We assume that raters who interpreted the case as a high expressor (TPS ≥50%) likely considered all positive cells as neoplastic cells, whereas raters who interpreted the case as negative (TPS <1%) interpreted all cells as nonneoplastic components (macrophages or mesothelial elements). Raters who interpreted the case with low expression levels (TPS 1%–49%) likely assessed only the cohesive component. Similarly, the case A7 showed neoplastic population both in groups and scattered, with specific membranous staining observed only in the latter (Figure 5). Finally, regarding the adequacy evaluation according to the routine criteria (<100 cells vs. >100 cells), a fair level of agreement was achieved, as indicated by a κ value of 0.36.

**TABLE 2:** Cases with the most discordant results among pathologists analyzed by identifying the three most common interpretation pitfalls.

	Nonspecific cytoplasmic staining	Membranous staining in nonneoplastic cells	Discohesive sample
TPS <1 vs. TPS 1%–49%			
A17	+	–	–
A22	–	+	–
B16	+	–	–
B20	–	+	–
B21	+	+	–
TPS 1%–49% vs. TPS ≥50%			
A1	+	–	+
A4	–	+	–
A23	+	–	–
B19	–	+	–
TPS <1% vs. TPS 1%–49% vs. TPS >50%			
B8	+	+	+
A7	–	+	–

Abbreviation: TPS, tumor proportion score.

## DISCUSSION

It is well established that cytological specimens provide an accurate assessment of PD‐L1 expression in most patients with NSCLC at both >1% and ≥50% cutoffs, when compared with histological material.^23^ This evidence is particularly relevant because CBs are used for PD‐L1 testing by a large proportion of pathologists worldwide.^24^ Nevertheless, interobserver variability is generally more pronounced in cytological samples than in biopsies and in surgical specimens confirming a more challenging interpretation of PD‐L1 expression for cytological samples.^11^, ^14^

Our study demonstrates an overall moderate agreement (κ = 0.57 for the two‐tier approach and κ = 0.49 for the three‐tier approach) for both PD‐L1 TPS interpretation. Better agreement was achieved within high positive (TPS ≥50%) and negative (TPS <1%) categories, whereas the lowest κ value was observed within TPS 1%–49% category. This distribution of interobserver agreement is in accordance with published data. Previous studies have highlighted lower raters of concordance in the low‐positive TPS category (1%–49%), suggesting that results in 1%–49% category are the least reliable PD‐L1 status assessment on cytology.^11^, ^13^, ^15^, ^16^ This phenomenon is comparable to what occurs in diagnostic cytopathology, in which moderate‐to‐good interobserver agreement is typically attainable in distinguishing between benign and malignant entities, whereas the diagnostic interpretation of indeterminate samples (atypical and suspicious categories) remains inconsistent. Indeed, high levels of variability reflect the difficulty in interpreting such cases, mainly ascribable to the overlapping cytomorphological features between clearly benign and malignant conditions.^25^ Similarly, the PD‐L1 evaluation of low expressor cases seems to be a significant challenge due to the presence of quantitative and qualitative intermediate features, mainly close to the clinically relevant cutoff. In this context, morphological pitfalls may greatly affect the assessment, such as the presence of nonspecific staining in the background and the difficulty in differentiating tumor cells from benign cells.^13^, ^14^, ^16^ Interestingly, we identified PD‐L1 expression in macrophages as one of the main pitfalls at all clinically relevant cutoffs. The challenge of distinguishing PD‐L1 expression in neoplastic cells versus macrophages was more pronounced in cases with dispersed tumor cells and/or well‐differentiated adenocarcinoma. These findings highlight the shortcomings of morphological evaluation alone and the value of immunocytochemistry, as confirmed by the fair agreement in evaluation of the neoplastic cell number (<100 vs. >100). In this setting, the immunohistochemical assessment of CD68 and TTF1 could be of assistance in the diagnostic process,^13^ however, in real life practice, the material may be so poor that the pathologist may need to preserve it for the predictive testing. Interestingly, our data demonstrate that, although increased in the two‐tier approach (<1%, >1%), the overall agreement achieved remains moderate.

Although the distribution of concordance values in this study is in line with previous evidence, we observed consistently lower κ values compared to prior literature data. Besides diagnostic criteria‐related issues, other factors may contribute to interobserver variability, including the variability in formal training received, the level and type of acquired experience and expertise of raters, as well as the number of raters. Studies involving a small number of highly skilled pathologists could provide biased results, showing higher agreement values than what would be expected in routine practice. Conversely, studies such as ours that include pathologists from different institutions with varying levels of experience, may more accurately reflects the heterogeneity of real‐life clinical practice.^26^ Moreover, increasing the number of raters dilutes the impact of outliers, resulting in a more representative and precise κ value. In the present study, 31 raters have been involved in the evaluation of 54 samples, for a total of 1674 observations representing what is, to the best of our knowledge, one of the largest interobserver concordance studies of PD‐L1 assessment on cytological material.

Limitations of this study include the lack of available information to participants regarding the results of ancillary immunohistochemical studies (i.e., CD68, TTF‐1) and the use of TMA instead of whole CB sections due to the practical constraints associated with sharing slides with raters; consequently, we did not test the real clinical practice in terms of evaluation of the adequacy of the samples. To overcome this issue, we asked for TPS even in cases with less than 100 cells.^18^, ^19^, ^20^ Moreover, because the evaluation of cytohistological concordance was out of the aim of our project, no data was produced; in fact, our raters assessed PDL1 expression only on TMA cores, and they did not have access to whole CB slides or to matched histological material. Finally, we did not perform a discussion of pitfalls with participants and a revision of discordant cases after a wash‐out period.

In conclusion, the overall moderate agreement observed in the SAMPLING project highlights that there is still room for improvement in inter‐pathologist agreement for PD‐L1 evaluation on cytological samples. Several actions could be taken to improve interobserver agreement among pathologists. Sample preparation procedures could be standardized and validated to ensure that samples are handled uniformly, reducing variability caused by differences in preparation techniques.^27^, ^28^ Moreover, the accuracy and consistency of PDL1 assessment on cytological samples could be significantly improved by a focused training, on the wake of what already reported in histology.^29^ In this regard, including cytological evaluation of PD‐L1 in external quality assessment (EQA) programs might help monitor proficiency and maintain high standards across laboratories. The use of CB could be practical for such proficiency testing programs. Finally, the application of machine learning‐derived image analysis tools in assisting pathologists for PD‐L1 assessment on histological material have been shown to be promising; literature data showed how some algorithms (e.g., Aitrox AI model) had performance for PD‐L1 expression assessment comparable to those of experienced pathologists.^30^ For this reason, validation of PD‐L1 expression by deep learning algorithms may assist pathologists in their assessment as potential “scoring assistant” or second opinion, reducing interobserver variability.^31^, ^32^ Despite the extensive research and progress on histological images, further research on cytological material is needed, and we suggest that cbTMA could help in validation of PD‐L1 assessment by deep learning algorithms on large scale studies.

## AUTHOR CONTRIBUTIONS

**Gennaro Acanfora**: Conceptualization, writing–review and editing, writing–original draft, data curation, and supervision. **Antonino Iaccarino**: Conceptualization, writing–original draft, writing–review and editing, data curation, and supervision. **Bruna Cerbelli**: Methodology, writing–review and editing, and formal analysis. **Claudio Di Cristofano**: Methodology, writing–review and editing, and project administration. **Claudio Bellevicine**: Conceptualization, writing–original draft, writing–review and editing, data curation, and supervision. **Massimo Barberis**: Writing–review and editing and formal analysis. **Emanuela Bonoldi**: Writing–review and editing and formal analysis. **Lukas Bubendorf**: Writing–review and editing and formal analysis. **Andreas Calaminus**: Writing–review and editing and formal analysis. **Severo Campione**: Writing–review and editing and formal analysis. **Sule Canberk**: Writing–review and editing and formal analysis. **Alberto Cavazza**: Writing–review and editing and formal analysis. **Giorgio Cazzaniga**: Writing–review and editing and formal analysis. **Obinna Chijioke**: Writing–review and editing and formal analysis. **Eduardo Clery**: Writing–review and editing and formal analysis. **Albino Eccher**: Writing–review and editing and formal analysis. **Marianne Engels**: Writing–review and editing and formal analysis. **Vincenzo Fiorentino**: Writing–review and editing and formal analysis. **Paolo Graziano**: Writing–review and editing and formal analysis. **Izidor Kern**: Writing–review and editing and formal analysis. **Ivana Kholova**: Writing–review and editing and formal analysis. **Jari Laatta**: Writing–review and editing and formal analysis. **Tania Labiano**: Writing–review and editing and formal analysis. **Martina Leopizzi**: Writing–review and editing and formal analysis. **Maria D. Lozano**: Writing–review and editing and formal analysis. **Rita Luis**: Writing–review and editing and formal analysis. **Elisabetta Maffei**: Writing–review and editing and formal analysis. **Alessandro Marando**: Writing–review and editing and formal analysis. **Maurizio Martini**: Writing–review and editing and formal analysis. **Elisabetta Merenda**: Methodology. **Marco Montella**: Writing–review and editing and formal analysis. **Allan Argueta Morales**: Writing–review and editing and formal analysis. **Michiya Nishino**: Writing–review and editing and formal analysis. **Fabio Pagni**: Writing–review and editing and formal analysis. **Paul Hofman**: Writing–review and editing and formal analysis. **Angelina Pernazza**: Writing–review and editing and formal analysis. **Sinchita Roy‐Chowdhuri**: Writing–review and editing and formal analysis. **Mauro Saieg**: Writing–review and editing and formal analysis. **Spasenija Savic Prince**: Writing–review and editing and formal analysis. **Momin T. Siddiqui**: Writing–review and editing and formal analysis. **Tajana Stoos‐Veic**: Writing–review and editing and formal analysis. **Margareta Strojan Fležar**: Writing–review and editing and formal analysis. **Dinka Sundov**: Writing–review and editing and formal analysis. **Paul VanderLaan**: Writing–review and editing and formal analysis. **Danijela Vrdoljak‐Mozetič**: Writing–review and editing and formal analysis. **Pio Zeppa**: Writing–review and editing and formal analysis. **Giancarlo Troncone**: Conceptualization, writing–original draft, writing–review and editing, data curation, and supervision. **Elena Vigliar**: Data curation, supervision, writing–review and editing, conceptualization, and writing–original draft.

## CONFLICT OF INTEREST STATEMENT

Paul VanderLaan reports consulting fees from Agilent Technologies, Inc, Galvanize Therapeutics, Intuitive Surgical, In, Ruby Robotics, and Veracyte Inc. Giancarlo Troncone reports consulting fees from Bayer, Merck Sharp and Dohme, Pfizer, and Roche Diagnostics GmbH. Lukas Bubendorf reports consulting fees from AbbVie, AstraZeneca, Bayer, Boehringer Ingelheim, Bristol‐Myers Squibb, Daiichi Sankyo Inc, Eli Lilly and Company, Johnson & Johnson Health Care Systems Inc, Merck, Pfizer, Systems Oncology, and Takeda Oncology; grant and/or contract funding from F. Hoffmann‐La Roche AG, Novartis, and Thermo Fisher Scientific; and stock in Hoffmann‐La Roche and Novartis. Severo Campione reports grant and/or contract funding from Menarini International. Michiya Nishino reports fees for professional activities from Elsevier and John Wiley & Sons. Spasenija Savic Prince reports consulting fees from AstraZeneca and Merck & Co.; fees for professional activities from AbbVie; and grant and/or contract funding from Dr. Arnold U. und Susanne Huggenberger‐Bischoff Stiftung zur Krebsforschung and Gilead Sciences (Gilead Foundation). The other authors declare no conflicts of interest.
